# Supplementary figures and images for: ReCSAI: recursive compressed sensing artificial intelligence for confocal lifetime localization microscopy
Source: BMC Bioinformatics. 2022 Dec 8;23:530. doi: 10.1186/s12859-022-05071-5 (PMC9732995; doi:10.1186/s12859-022-05071-5)

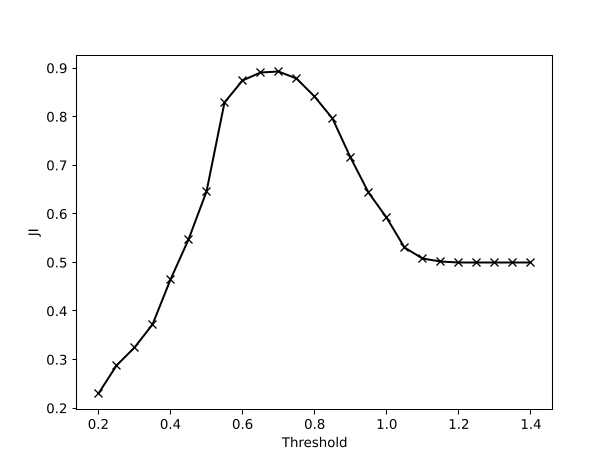

Supplement: Supplementary file 1 — Additional file 1. Fig. S1. Reconstruction threshold. To identify the optimal threshold to reconstruct localisations from feature space, we tested the influence of the parameter on our validation data. It can be seen that the Jaccard Index peaks for a value of 0.7. [file 12859_2022_5071_MOESM1_ESM.png]

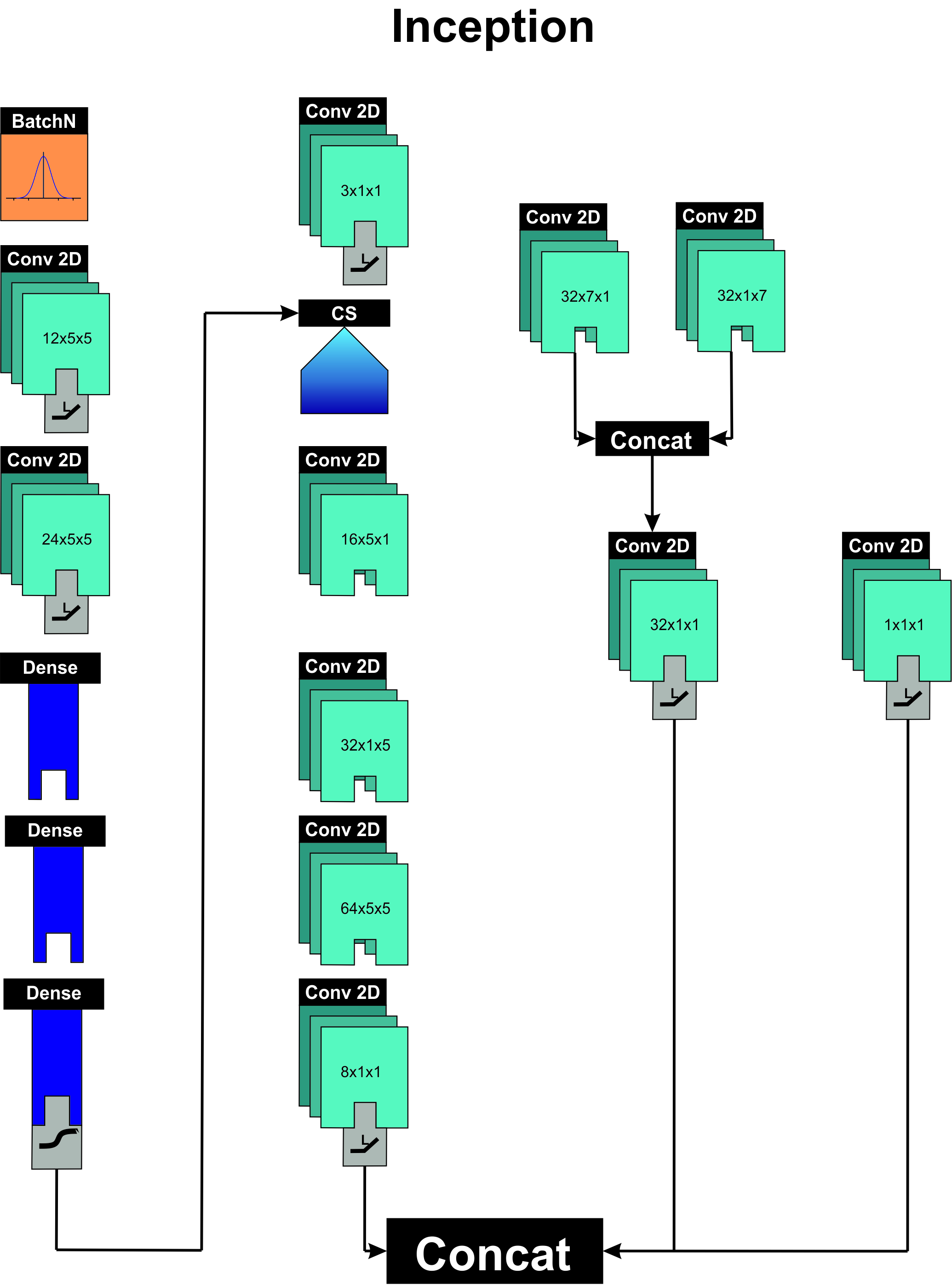

Supplement: Supplementary file 2 — Additional file 2. Fig. S2. Inception building block. This building block is derived from the inception network [20]. The input is processed in 4 different paths (from left to right). We estimate the compressed sensing parameter $\lambda$λ with a conventional CNN. The input is processed by a bottleneck layer, followed by the compressed sensing layer. Several convolutional layers restore the original image size. A feature detector applies asymmetric filters from different directions. A pass-through only applies an activation function, passing forward the original image. [file 12859_2022_5071_MOESM2_ESM.jpg]

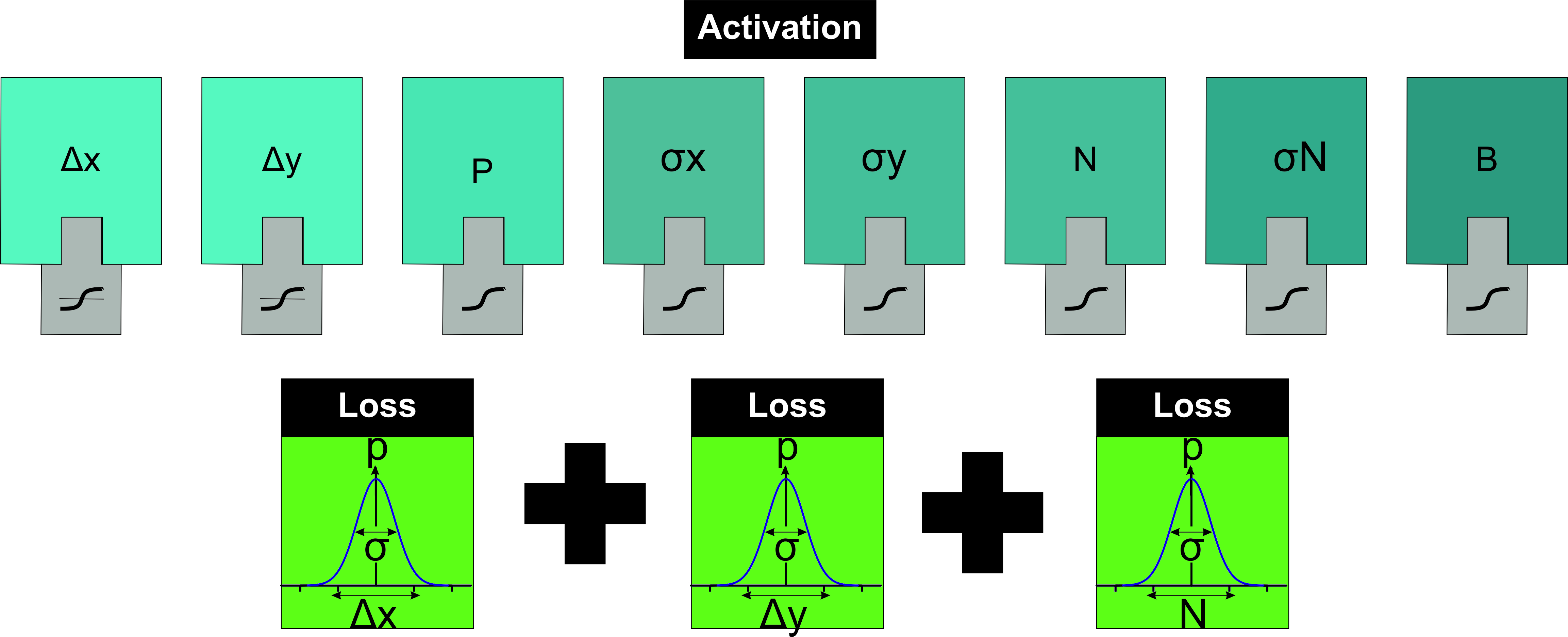

Supplement: Supplementary file 3 — Additional file 3. Fig. S3. Activations of the output layer. The relative positions $\Delta x$Δx and $\Delta y$Δy are fed into a Gaussian mixture model with the local probability p and the positional uncertainties $\sigma _x$σx and $\sigma _y$σy. The estimated intensity N is included into the localisation loss. While $\Delta x$Δx and $\Delta y$Δy are tanh activated, all other components are sigmoid activated. [file 12859_2022_5071_MOESM3_ESM.jpg]

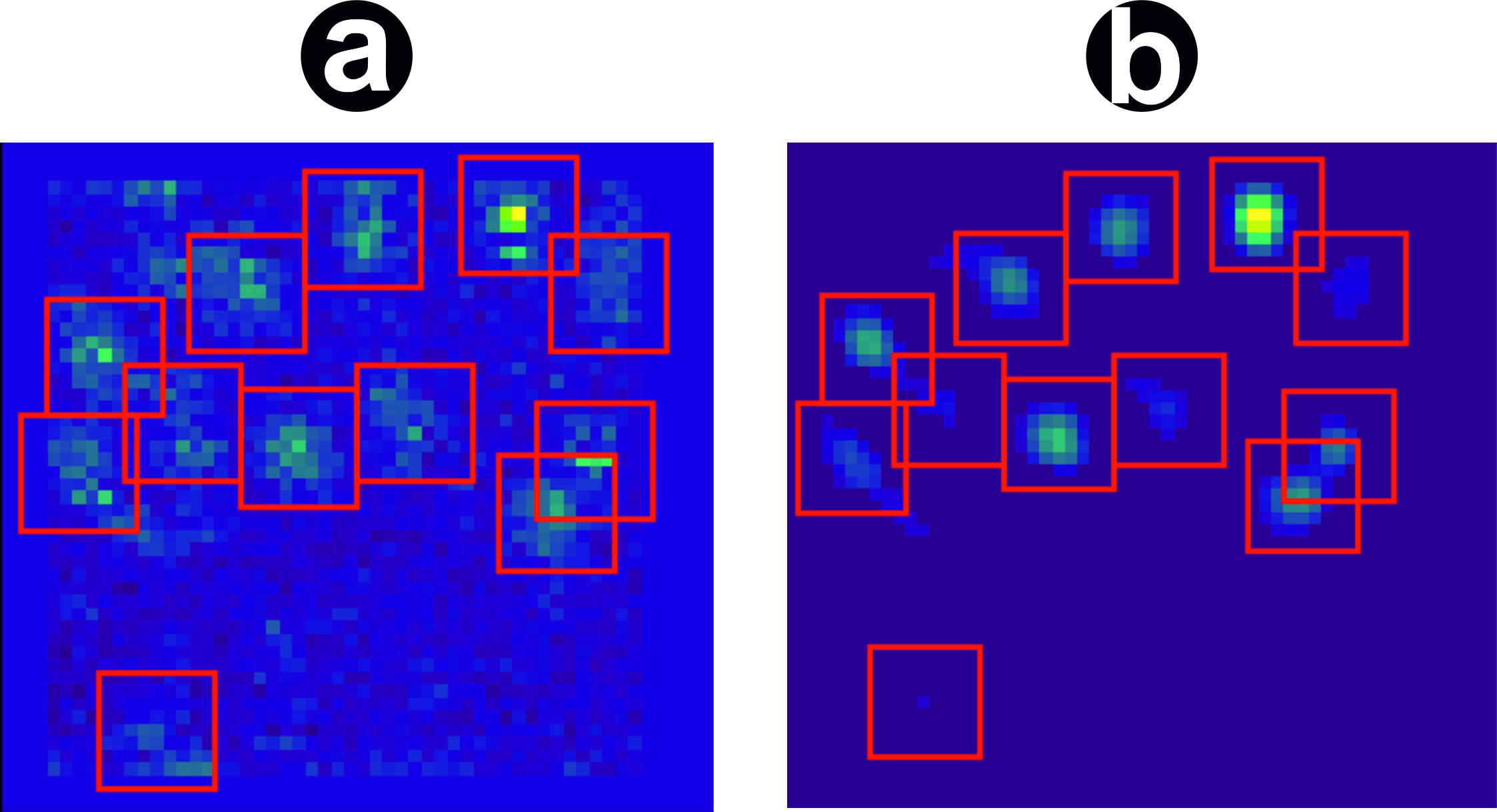

Supplement: Supplementary file 4 — Additional file 4. Fig. S4. Wavelet filter bank peak detection on an example frame of a FLIMbee measurement. The input image a is deconstructed with a wavelet filter bank, trained to extract frequencies resembling a PSF. A threshold is applied before reconstructing to generate a denoised image b. Potential emitters are identified with a local maximum detection. The detected peaks are marked with red rectangles. [file 12859_2022_5071_MOESM4_ESM.jpg]
